# Supplementary figures and images for: Symbiotic bacteria Sodalis glossinidius, Spiroplasma sp and Wolbachia do not favour Trypanosoma grayi coexistence in wild population of tsetse flies collected in Bobo-Dioulasso, Burkina Faso
Source: BMC Microbiol. 2024 Sep 28;24:373. doi: 10.1186/s12866-024-03531-x (PMC11437622; doi:10.1186/s12866-024-03531-x)

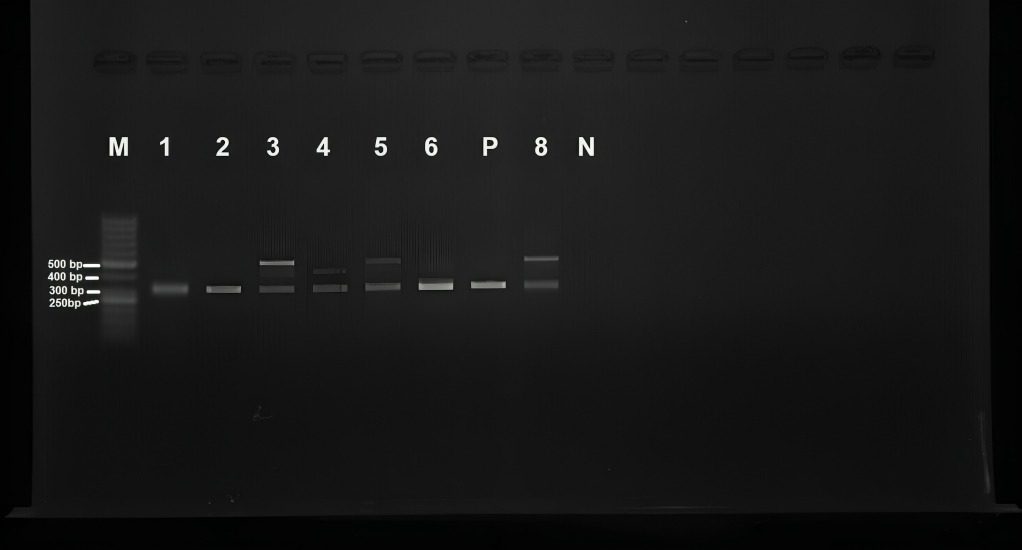

Supplement: Supplementary file 1 — Supplementary Material 1 [file 12866_2024_3531_MOESM1_ESM.jpg]
